# Seasonal Metagenomic Survey of Pathogenic Microorganisms in Non-Human Primates in Mayanghe National Nature Reserve, China

**DOI:** 10.3390/pathogens14121237

**Published:** 2025-12-04

**Authors:** Ping Liu, Dan Wang, Fan Zhang, Tao Wang, Jialiang Han, Qixian Zou

**Affiliations:** 1 Academic Affairs Office, Sichuan Nursing Vocational College, Chengdu 610100, China; 2 Department of Internal Medicine, Public Health Clinical Center of Chengdu, Chengdu 610021, China; 3 Mayanghe National Nature Reserve Administration, Tongren 554400, China; 4 School of Basic Medical Sciences, Chengdu University, Chengdu 610106, China; 5 Office of Academic Affairs, Chengdu University, Chengdu 610106, China

**Keywords:** non-human primates, metagenomics, seasonal variation, pathogenic microorganisms

## Abstract

Understanding the diversity of pathogenic microorganisms in wild primates is essential for assessing their health and zoonotic risks. In this study, metagenomic sequencing was applied to investigate the composition and seasonal dynamics of potential pathogenic microorganisms in the feces of *François’ langurs*. A total of 77 potential pathogenic taxa were identified, mainly belonging to *Bacillota* and *Pseudomonadota*. The most abundant genera were *Streptococcus*, *Staphylococcus*, *Salmonella*, *Listeria*, and *Pseudomonas*, while dominant species included *Staphylococcus aureus*, *Streptococcus pneumoniae*, *Salmonella enterica*, *Listeria monocytogenes*, and *Escherichia coli*. Significant seasonal differences were detected in both α- and β-diversity indices, with higher microbial diversity in spring and distinct community structures across seasons. Several genera and species, including *Vibrio*, *Chlamydia*, *Mycobacteroides*, *Vibrio cholerae*, *Yersinia enterocolitica*, *Chlamydia trachomatis*, and *Mycobacteroides abscessus*, showed marked seasonal fluctuations. The findings reveal that the pathogenic microbial community of *François’ langurs* is strongly shaped by seasonal environmental factors. The detection of multiple zoonotic pathogens suggests a potential risk of cross-species transmission, providing valuable baseline data for primate disease ecology and conservation health management.

## 1. Introduction

Diseases are major factors influencing the survival, reproduction, and long-term viability of wildlife populations [1,2]. Wild animals are susceptible to a wide range of pathogens, including viruses, bacteria, fungi, and parasites [3,4]. These pathogens can be transmitted through the fecal-oral route, posing potential risks to both wildlife and human health. Studies have identified zoonotic pathogens such as *Salmonella* spp., *Escherichia coli*, and *Giardia* spp. in the feces of various wildlife species [5,6,7]. Non-human primates are of particular interest due to their close genetic relationship with humans, which facilitates the potential transmission of pathogens between species [8,9]. The findings underscore the importance of monitoring fecal pathogens in primate populations to assess health risks and inform conservation and public health strategies. Previous research has documented the presence of several zoonotic pathogens in the feces of wild primates, including enteric bacteria (*Salmonella* spp., *Escherichia coli*), protozoan parasites (*Giardia* spp., *Cryptosporidium* spp., *Entamoeba* sp.), and viruses with zoonotic potential (e.g., adenoviruses, simian foamy viruses) [10,11].

Metagenomic sequencing has become a powerful approach for investigating pathogens in wildlife, offering a culture-independent method that enables the simultaneous detection of bacteria, viruses, fungi, and parasites directly from environmental or fecal samples [12,13,14,15]. Metagenomic sequencing is not only a key tool for pathogen surveillance but also a framework for assessing cross-species transmission risks and ecosystem health. Traditional diagnostic techniques, metagenomics can identify both known and previously uncharacterized microorganisms, thereby broadening our understanding of pathogen diversity and evolution [16,17,18]. Recent applications in wildlife disease ecology have revealed hundreds of novel viral and bacterial taxa from fecal samples of diverse hosts, including herbivores and primates, many of which carry zoonotic potential [19,20]. For primates, metagenomic analyses of feces have uncovered enteric viruses, gut parasites, and microbial dysbiosis associated with anthropogenic disturbance, highlighting both conservation and public health relevance [9,21,22,23].

*François’ langur* (*Trachypithecus francoisi*) is a folivorous primate, primarily inhabiting karst limestone forests in southern China and northern Vietnam [24,25]. It is classified as Endangered on the IUCN Red List and as a Class I protected species in China [26] (IUCN, 2023). The habitat of *François’ langurs* is fragmented and increasingly isolated due to habitat loss and human disturbances [25,27,28]. The Mayanghe National Nature Reserve in Guizhou Province represents one of the most important strongholds for this species in China [24,29,30]. Landscape fragmentation in this region has resulted in highly dispersed habitat patches with complex boundaries, reducing population connectivity and potentially limiting gene flow [31,32]. Seasonal fluctuations in food availability further challenge the health and resilience of these populations [29], with studies indicating that gut microbial diversity varies significantly across seasons, reflecting changes in diet and habitat conditions [33,34]. These insights into habitat and ecological constraints, systematic data on the pathogens and microorganisms carried by wild *François’ langurs* remain scarce, highlighting a critical knowledge gap for disease surveillance and conservation planning.

In this study, we collected fecal samples from *François’ langurs* in the Mayanghe National Nature Reserve across four seasons and performed metagenomic sequencing to characterize their gut microbial and pathogenic communities. The objectives were to: (i) identify potential pathogens carried by *François’ langurs*; (ii) assess seasonal variations in microbial diversity. The findings will provide essential baseline data for monitoring the health of *François’ langurs* and conservation management of this endangered primate.

## 2. Methods

### 2.1. Study Area

This study was carried out in the Mayanghe National Nature Reserve (MNNR) (28°37′30″–28°54′20″ N, 108°3′58″–108°19′45″ E), located in the northeast of Guizhou Province, southwestern China, covering an area of 311.13 km^2^. The reserve was established in 1987 mainly for the protection of *François’ langurs* and their karst forest habitat [29]. The altitude ranges from 280 to 1441 m, with a typical subtropical monsoon climate characterized by warm, humid, and rainy conditions and distinct seasonal variation in temperature and precipitation [35]. The vegetation types include evergreen broadleaf forest, coniferous forest, coniferous–broadleaf mixed forest, deciduous broadleaf forest, bamboo forest, and shrubland [36]. Totaling 500–600 individuals, exist in the reserve [37].

### 2.2. Sample Collection

The study group of *François’ langurs* consisted of 16 individuals, including 11 adults and 5 infants, inhabiting the southwestern part of MNNR. The langurs are well habituated to human observers. Their natural diet mainly consists of leaves, fruits, flowers, and buds [29]. *François’ langurs* usually sleep in limestone caves or on cliff platforms and defecate before leaving the site in the morning.

Prior to sampling, the sleeping sites of the langur group were observed and recorded. Fresh fecal samples were collected in the morning under the cliffs after the langurs had left. To minimize duplicate sampling from the same individual, samples were collected at intervals of more than 3 m. Only the inner portion of the feces, which had not been in contact with the external environment, was taken using sterile gloves and germ-free tools such as bamboo skewers. Each sample (3–5 g) was immediately transferred into a sterile, labeled collection tube and preserved in RNAlater solution (QIAGEN, Valencia, CA, USA) to stabilize nucleic acids. Samples were then stored at −80 °C until further processing. Sampling was conducted in October 2023, January 2024, May 2024, and July 2024, representing the four seasons (autumn, winter, spring, and summer). In total, 24 fecal samples were collected for metagenomic analysis, Samples were collected randomly, and individual identity could not be determined in the field; Paired samples from specific individuals were not obtained. The fecal samples used in this study were collected from the same *François’ langur* group as described in our previously published work [34]. The present study focuses specifically on the seasonal dynamics of pathogenic communities, which were not addressed in the earlier analysis.

### 2.3. DNA Extraction, Library Preparation, and Sequencing

Genomic DNA (gDNA) was extracted from *François’ langur* fecal samples using the BIOMICS DNA Microprep Kit (Cat# D4301, Zymo Research, Tustin, CA, USA) according to the manufacturer’s instructions. DNA quality and integrity were assessed by 0.8% agarose gel electrophoresis and an Agilent 2100 Bioanalyzer (Agilent Technologies, Santa Clara, CA, USA). DNA concentration was quantified using the PicoGreen dsDNA assay on a Tecan F200 microplate reader [38]. High-quality gDNA was used for library construction with the NEBNext Ultra II DNA Library Prep Kit for Illumina (Cat# E7645, New England Biolabs, Ipswich, MA, USA) and NEBNext Multiplex Oligos (Cat# E7600). Fragmentation, end repair, adaptor ligation, and PCR enrichment were carried out following the manufacturer’s protocol. Library quality and fragment size were examined using the Agilent 2100 Bioanalyzer, and concentrations were determined by quantitative PCR (qPCR). Qualified libraries were sequenced on the Illumina NovaSeq 6000 platform with the NovaSeq 6000 S4 Reagent Kit (300 cycles, Cat# 20039236, Illumina, San Diego, CA, USA), producing 150 bp paired-end reads (PE150). All experiments were conducted under strict quality control to ensure the reliability and reproducibility of metagenomic data.

### 2.4. Data Processing and Quality Control

Raw reads were quality-filtered using Trimmomaticto remove adapters and low-quality bases [39]. Reads shorter than 100 bp or with average quality scores below Q15 were discarded. A Q15 cutoff was chosen to retain sufficient read coverage for downstream metagenomic assembly and analysis, given the potential fragmentation and variable quality of fecal-derived DNA, while preliminary analyses with a more stringent Q30 cutoff produced highly consistent taxonomic and functional profiles. To eliminate host contamination, filtered reads were aligned to the *François’ langur* reference genome using BWA (version 0.7.19) [40], and unmapped reads were retained with SAMtools for downstream analyses [41]. Clean reads were assembled de novo using SPAdes (v3.15.3) in metagenomic mode [42], and assembly quality was evaluated with QUAST (v5.0.2) [43]. Open reading frames (ORFs) were predicted using Prodigal (v2.6.3) [44].

### 2.5. Gene Catalog Construction and Functional Annotation

Predicted genes from all samples were clustered into a non-redundant gene catalog using MMseqs2 (version 15.6f452) with 95% sequence identity and 90% alignment coverage thresholds [45]. The longest sequence in each cluster was selected as the representative. Quality-filtered reads were mapped to the catalog using BBMap (version 39.01), and gene abundances were normalized as TPM or RPKM values [46]. Representative genes were annotated against multiple databases, including NR, UniProt, KEGG, CAZy CARD, VFDB, and PHI-base, to explore microbial diversity, functional profiles, and potential pathogenicity [47,48].

### 2.6. Microbial Diversity and Statistical Analysis

Taxonomic and diversity analyses were performed in R (v4.0.5). Alpha diversity indices were calculated using the vegan and picante packages, and group comparisons were tested with the Wilcoxon rank-sum test and agricolae for post hoc analysis. Beta diversity was estimated using Bray–Curtis, Jaccard, and UniFrac distances, and visualized via PCA, PCoA, and NMDS. Group differences were assessed with ANOSIM and PERMANOVA (adonis function in vegan). Differentially abundant taxa among seasons were identified using LEfSe (Galaxy platform, The Pennsylvania State University, University Park, PA, USA) [12]. Taxonomic assignments were based on the SILVA 138 database [49], and visualization was completed in R (version 4.2.2) and Python (v3.7.4).

## 3. Results

### 3.1. Sequencing Quality

Metagenomic sequencing of 24 François’ langur fecal samples generated ~1.99 billion raw paired-end reads (32.79–54.21 million per sample; Supplementary Table S1). After adapter trimming, Q15 quality filtering (Trimmomatic), and removal of host reads via BWA, 1.98 billion high-quality reads were retained (32.68–54.08 million per sample), ad-dressing reviewer concerns on per-sample read counts. Taxonomic profiles were inferred directly from clean reads using the k-mer–based classifier Kraken2, which provides high-resolution assignments without assembly, explicitly responding to the reviewer’s question on taxonomy determination. For gene prediction and functional annotation, de novo assembly with SPAdes generated 63,766–148,962 contigs per sample (≥1 kb; N50 2.8–11.2 kb; average 4.3 kb). Gene prediction yielded 15.3 million coding sequences, clus-tered into 1.05 million non-redundant genes, forming a comprehensive gene catalog. 

### 3.2. Composition of Potential Pathogenic Microorganisms of François’ langur

Potential pathogenic microorganisms in *François’ langur* were identified by aligning metagenomic sequencing data against the PHI-base database (http://www.phi-base.org/). After excluding plant and insect pathogens, a total of 77 potential pathogenic microorganisms were detected, spanning 8 Phylum, 13 classes, 25 orders, 38 families, and 49 genuses. In this study, genus and species-level data were selected for subsequent analysis of the composition of pathogenic microorganisms across different samples.

**Figure 1:**
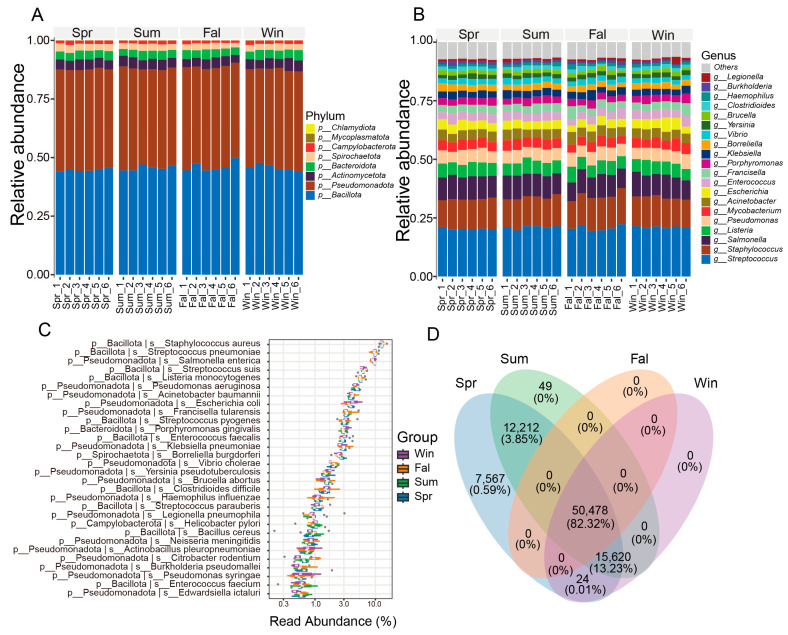
Composition and distribution of potential pathogenic microorganisms across seasons. (**A**) Bar plot showing the relative abundance of major taxa at the phylum level. (**B**) Bar plot showing the relative abundance of dominant taxa at the genus level. (**C**) Box plot illustrating the relative abundance of high-abundance pathogenic species among different seasonal groups. (**D**) Venn diagram showing the numbers of shared and unique OTUs among groups. The numbers represent OTU counts, while the percentages in parentheses indicate the proportion of total sequences contributed by these OTUs relative to the total number of sequences from all OTUs used in this analysis.

At the phylum level, the pathogenic microorganisms community of *François’ langur* displayed no seasonal variation (Figure 1A). *Bacillota*, *Pseudomonadota*, *Actinomycetota*, *Bacteroidota* were the dominant phyla in four seasons. At the genus level, *Streptococcus, Staphylococcus*, *Salmonella*, *Listeria* and *Pseudomonas* were the top five taxa (Figure 1B). The dominant taxa belonged to *Bacillota*, mainly *Streptococcus*, *Staphylococcus*, *Listeria*, *Enterococcus*, and *Bacillus*, which are common gut or mucosal bacteria with opportunistic pathogenic potential. Members of *Pseudomonadota*, including *Salmonella*, *Escherichia*, *Klebsiella*, *Pseudomonas*, and *Acinetobacter*, were also abundant, reflecting enteric and environmental origins. Other detected genera such as *Brucella*, *Yersinia*, *Burkholderia*, *Vibrio*, and *Haemophilus* indicate possible zoonotic relevance.

At the species level, *Staphylococcus aureus*, *Streptococcus pneumoniae*, *Salmonella enterica*, *Streptococcus suis*, *Listeria monocytogenes* were the top five species in four seasons (Figure 1C). The community was dominated by members of the phylum Bacillota, including *Staphylococcus aureus*, *Streptococcus pneumoniae*, and *Listeria monocytogenes*, which are typical opportunistic pathogens causing respiratory and systemic infections in mammals. Other Bacillota species, such as *Enterococcus faecalis*, *E. faecium*, and several *Streptococcus* taxa (*S. pyogenes*, *S. agalactiae*, *S. suis*), were also prevalent. Among *Pseudomonadota*, *Escherichia coli*, *Salmonella enterica*, *Klebsiella pneumoniae*, and *Pseudomonas aeruginosa* were dominant, reflecting enteric and environmental bacterial sources with known zoonotic potential. Gram-negative pathogens, including *Acinetobacter baumannii* and *Haemophilus influenzae*, indicated possible respiratory involvement. Several zoonotic agents, such as *Brucella abortus*, *Leptospira interrogans*, and *Coxiella burnetii*, were detected at lower abundance, implying potential cross-species transmission.

A Venn diagram was generated based on the OTU (Operational Taxonomic Unit) abundance to compare the shared and unique microbial taxa among different seasonal groups (Figure 1D). A total of 50,478 OTUs (82.32%) were shared among all four seasons. Spring contained the highest number of unique OTUs (7567, 0.59%), while no unique OTUs were observed in fall or winter. In addition, 12,212 OTUs (3.85%) were shared between spring and summer, and 15,620 OTUs (13.22%) were shared among spring, summer, and winter. Other combinations contributed negligibly to the total diversity.

### 3.3. Analysis of α- and β-Diversity

**Figure 2:**
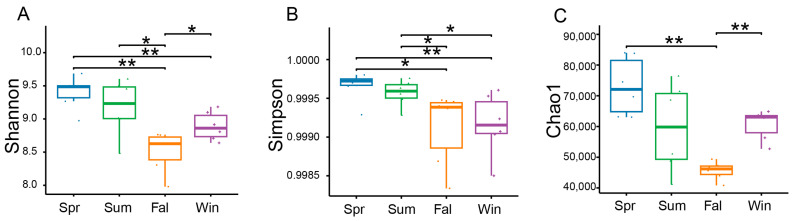
Seasonal variation in the α-diversity of potential pathogenic microorganisms in *François’ langurs*. Box plots illustrating species richness and diversity across samples from different seasons. α-diversity was evaluated using the Shannon (**A**), Simpson (**B**), and Chao1 (**C**). * *p* < 0.05, ** *p* < 0.01 (Wilcoxon rank-sum test).

Based on sequencing depth, the potential pathogenic microbial communities exhibited high coverage, four α-diversity indices—including the Shannon index, Simpson index and Chao1—were calculated to assess the diversity and richness of potential pathogenic microorganisms. Seasonal variations in α-diversity were evaluated using linear mixed models. Significant seasonal differences were observed in the α-diversity of potential pathogenic microbial communities. The Shannon index showed clear seasonal fluctuations, with significantly higher diversity in spring samples compared with those collected in fall (Figure 2A). The Simpson index revealed higher diversity in spring relative to fall and winter (Figure 2B). The Chao1 index also varied across seasons, with winter samples exhibiting greater species richness than those from fall (Figure 2C).

**Figure 3:**
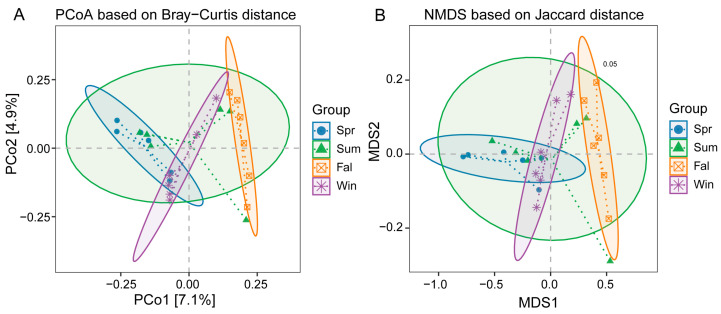
β-diversity analysis of potential pathogenic microorganisms in *François’ langurs* across different seasons. (**A**) Principal Coordinate Analysis (PCoA) plot based on Bray–Curtis distances among the four seasonal groups. (**B**) Non-metric multidimensional scaling (NMDS) ordination based on Bray–Curtis distances with group ellipses indicating clustering patterns among seasonal groups.

β-diversity analyses were conducted based on species-level relative abundance profiles. Bray–Curtis dissimilarity and both weighted and unweighted UniFrac distance metrics were calculated using QIIME2. Principal Coordinate Analysis (PCoA) showed clear separation among samples from different seasons (Figure 3A). The first two principal coordinates explained 7.1% and 4.9% of the total variation, respectively. Samples from spring and summer clustered closely together, while those from fall and winter were clearly separated, indicating significant seasonal shifts in community composition. The NMDS analysis based on Bray–Curtis distance showed clear separation of microbial communities among the four seasons (Stress = 0.05) (Figure 3B). Spring, summer, fall, and winter samples formed distinct clusters with minimal overlap, indicating strong seasonal variation in community composition.

PERMANOVA results confirmed significant differences in community structure among seasons (Table 1), the composition of potential pathogenic microorganisms differed significantly between spring and fall (R^2^ = 0.123, *p* = 0.004), spring and winter (R^2^ = 0.099, *p* = 0.023), summer and fall (R^2^ = 0.099, *p* = 0.040), fall and winter (R^2^ = 0.106, *p* = 0.005). No significant differences were observed between spring and summer (*p* = 0.069) or summer and winter (*p* = 0.248).

### 3.4. Differential Species Analysis

**Figure 4:**
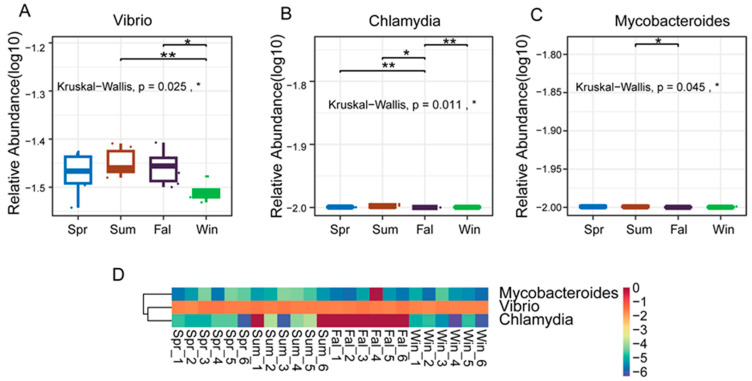
Differential analysis of dominant pathogenic genera in *François’ langurs* across seasons. (**A**,**B**,**C**) Boxplots showing the relative abundance and pairwise seasonal comparisons of the top 10 differential genera. (**A**) *Vibrio*, (**B**) *Chlamydia* and (**C**) *Mycobanteroides* exhibited significant seasonal variation in abundance. (**D**) Heatmap illustrating the relative abundance patterns of the top 5 differential genera across all samples. Each column represents an individual sample, and each row represents a genus. Color intensity indicates relative abundance levels. * *p* < 0.05, ** *p* < 0.01.

The study applied the non-parametric Kruskal–Wallis (KW) rank-sum test to evaluate differences in species abundance among groups. Initially, a global KW test was conducted for all species to identify those exhibiting significant overall differences. Subsequently, the top abundant species were subjected to pairwise comparisons between groups. Relative abundance boxplots and heatmaps were generated to visualize the distribution of dominant differential species across groups. At the genus level, three pathogenic genera—*Vibrio*, *Chlamydia*, and *Mycobacteroides*—exhibited significant seasonal differences in relative abundance (*p* < 0.05). *Vibrio* showed nearly significant variation between summer and winter (*p* < 0.01) (Figure 4A), and a significant difference between fall and winter (*p* < 0.05). *Chlamydia* displayed nearly significant differences between spring and fall (Figure 4B), fall and winter (*p* < 0.01), while a significant difference was observed between summer and autumn (*p* < 0.05). *Mycobacteroides* demonstrated a significant difference between summer and autumn (*p* < 0.05). As illustrated in the boxplots and heatmaps (Figure 4C), these genera exhibited distinct seasonal abundance patterns. Vibrio tended to be more abundant in the summer and fall, whereas Chlamydia and Mycobacteroides showed notable fluctuations between transitional and extreme temperature periods.

**Figure 5:**
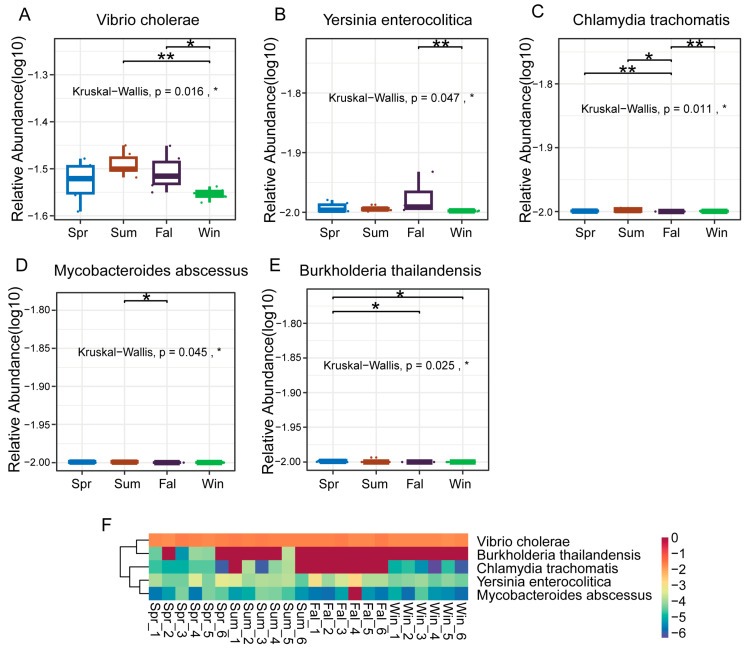
Differential analysis of dominant pathogenic species in *François’ langurs* across seasons. (**A**–**E**) Boxplots showing the relative abundance and seasonal differences of the top 10 differential species. (**A**) *Vibrio cholerae*, (**B**) *Yersinia enterocolitica*, (**C**) *Chlamydia trachomatis*, (**D**) *Mycobacteroides abscessus*, and (**E**) *Burkholderia thailandensis* exhibited significant seasonal variation in relative abundance. (**F**) Heatmap showing the relative abundance patterns of the top 50 differential species across all samples. Each column represents an individual sample, and each row represents a species. Color intensity indicates relative abundance levels. * *p* < 0.05, ** *p* < 0.01.

At the species level, five pathogenic species (*Vibrio cholerae*, *Yersinia enterocolitica*, *Chlamydia trachomatis*, *Mycobacteroides abscessus*, and *Burkholderia thailandensis*) showed significant seasonal differences in relative abundance. *Vibrio cholerae* exhibited a significant difference between summer and winter, and between fall and winter (Figure 5A). *Yersinia enterocolitica* exhibited a highly significant difference between fall and winter (*p* < 0.01) (Figure 5B). *Chlamydia trachomatis* showed nearly significant differences between spring and fall, and between autumn and winter (*p* < 0.01) (Figure 5C), while *Mycobacteroides abscessus* demonstrated a significant difference between summer and autumn (*p* < 0.05) (Figure 5D). Boxplots and heatmaps further confirmed these seasonal trends (Figure 5F), indicating that *Vibrio cholerae* and *Yersinia enterocolitica* were more abundant in warmer or transitional seasons, whereas *Chlamydia trachomatis* and *Mycobacteroides abscessus* fluctuated markedly between seasons.

## 4. Discussion

In this study, a rich diversity of pathogenic bacterial genera and species in *François’ langurs* fecal samples. The dominant genera belonged to *Bacillota* and *Pseudomonadota*, mainly *Streptococcus*, *Staphylococcus*, *Listeria*, *Enterococcus*, *Salmonella*, *Escherichia*, *Klebsiella*, and *Pseudomonas*. At the species level, notable taxa included *Staphylococcus aureus*, *Streptococcus pneumoniae*, *Escherichia coli*, *Salmonella enterica*, *Listeria monocytogenes*, *Klebsiella pneumoniae*, *Pseudomonas aeruginosa*, *Acinetobacter baumannii*, *Brucella abortus*, Y*ersinia enterocolitica*, *Coxiella burnetii*, *Leptospira interrogans*, and *Mycobacteroides abscessus*. These bacteria are commonly found as gut or mucosal residents but may act as opportunistic pathogens under conditions of physiological stress or immune suppression [50].

The presence of *Streptococcus* and *Staphylococcus* genera, especially *S. pneumoniae* and *S. aureus*, suggests possible colonization or transmission of respiratory–associated bacteria. *Streptococcus* is a genus that includes both commensal and pathogenic species and is commonly found in the gastrointestinal tract and on the mucosal surfaces of animals [51,52]. In nonhuman primates, *Streptococcus pneumoniae* has been reported in some health and disease studies [9,53,54,55]. Lower-abundance genera, including *Brucella*, *Yersinia*, *Coxiella*, and *Leptospira*, are notable for their role in livestock and wildlife infections. Their detection indicates potential cross-species exposure in shared habitats, highlighting the importance of monitoring primate populations as sentinels for emerging zoonoses.

At both the genus and species levels, *Vibrio*, *Chlamydia*, and *Mycobacteroides* exhibited significant seasonal differences, with *Vibrio cholerae* and *Yersinia enterocolitica* being more abundant in warmer or transitional seasons. These patterns are consistent with the known ecology of pathogens, which thrive in humid or nutrient-rich conditions, primates housed in a sanctuary represent an intermediate microbiome state between wild and captive [56,57,58]. *Chlamydia trachomatis* and *Mycobacteroides abscessus* showed fluctuations across temperature extremes, possibly related to host immune modulation [59,60]. The presence of these zoonotic bacteria suggests a potential risk of cross-species infection. Although cross-species transmission between humans and macaques is well documented, similar mechanisms may also occur in *François’ langurs* sharing habitats with humans. Seasonal environmental changes may thus enhance opportunities for pathogen transmission, as observed in other non-human primates and wildlife systems, including wildlife zoonotic and vector-borne EIDs originating at lower latitudes where reporting effort is low [15,61,62]. Continuous surveillance of these pathogens is essential for understanding their ecological drivers and for mitigating potential zoonotic risks to both primate and human health.

Significant seasonal variation was observed in the diversity and composition of potential pathogenic microorganisms in the fecal microbiota of *François’ langurs*. The higher α-diversity in spring suggests that environmental and dietary factors during this period promote microbial richness, consistent with findings in other wild primates showing seasonal shifts linked to food availability and host physiology [63,64,65,66,67]. β-diversity analyses further revealed clear separation among seasons, indicating that the structure of pathogenic microbial communities changed dynamically over time. These temporal fluctuations likely reflect ecological factors such as temperature, humidity, and resource variation, which can influence bacterial transmission and survival in the environment [68,69,70]. These results reveal distinct seasonal trends in the abundance of key pathogenic genera, implying that shifts in environmental parameters and ecological interactions across different times of the year may play a critical role in shaping pathogen dynamics.

Climatic and ecological factors may influence the occurrence of zoonotic pathogens in wild primates. Seasonal shifts in temperature and resource availability can influence host physiology and gut microbial stability in wild primates. In our study, significant seasonal differences in α- and β-diversity and in the relative abundances of pathogenic taxa (e.g., *Vibrio*, *Chlamydia*, *Mycobacteroides*; *Vibrio cholerae*, *Yersinia enterocolitica*, *Chlamydia trachomatis*) suggest that environmental variation may modulate pathogen dynamics. Climatic stress is known to weaken immune defenses and promote dysbiosis, increasing infection risk. Such instability may affect health and survival in wild primates, especially in species with small or fragmented populations [66,67,71,72].

Given the frequent overlap between *François’ langurs* habitats and human activity areas, such microbial fluctuations could elevate the risk of cross-species transmission. Seasonal monitoring of pathogen communities can thus provide valuable early indicators of disease emergence and help guide targeted prevention strategies. From a conservation perspective, integrating microbiome-based surveillance into long-term wildlife management programs could enhance early detection of pathogenic threats. Measures such as reducing human–primate contact, maintaining clean water sources, and improving waste management in habitats are essential to minimize zoonotic spillover risks. Microbiome monitoring provides a non-invasive and sensitive tool for assessing ecosystem health, offering new insights for wildlife conservation in changing environments.

Fecal samples were collected from a single social group, and individual identities could not be determined. The findings primarily reflect group-level patterns in pathogen prevalence, while individual-level variation remains to be explored. Future studies incorporating microsatellites or SNP analyses could help reconstruct individual identities and provide more detailed insights. Seasonal changes in food availability and social contact may also vary among groups or years, potentially influencing microbial and pathogen dynamics.

The analyses focused exclusively on DNA-based metagenomics, which allowed characterization of bacterial and DNA viral communities but does not allow conclusions about RNA viruses such as Zika or other arboviruses. Reads shorter than 100 bp or with average quality scores below Q15 were discarded to retain sufficient coverage for metagenomic assembly. Preliminary analyses using a more stringent Q30 cutoff confirmed that taxonomic and functional profiles were highly consistent, supporting the robustness of the results. The study concentrated on bacterial pathogens and excluded plant and insect pathogens, such as Aspergillus and arboviruses, which may also contribute to gut microbial composition. Future work involving multiple groups, long-term sampling, RNA-based analyses, and non-bacterial pathogens will provide a more comprehensive understanding of gut pathogen diversity and seasonal dynamics in *François’ langurs*.

## 5. Conclusions

The study presents the first metagenomic characterization of potential pathogenic microorganisms in the fecal microbiota of *François’ langurs* across four seasons. A total of 77 potential pathogenic taxa were identified, mainly belonging to *Bacillota* and *Pseudomonadota*, with *Streptococcus*, *Staphylococcus*, *Salmonella*, *Listeria*, and *Pseudomonas* as dominant genera. Both α- and β-diversity analyses revealed clear seasonal variations, with higher microbial diversity in spring and distinct community structures between fall and winter. Several pathogenic taxa, including *Vibrio cholerae*, *Yersinia enterocolitica*, *Chlamydia trachomatis*, and *Mycobacteroides abscessus*, exhibited marked seasonal fluctuations, reflecting the influence of environmental changes on pathogen dynamics in wild primates. The detection of multiple zoonotic microorganisms highlights the potential risk of cross-species transmission and underscores the need for long-term monitoring to better understand host–microbe–environment interactions and support conservation of endangered primate populations.

## Figures and Tables

**Table 1:** Results of PERMANOVA (Permutational Multivariate Analysis of Variance) based on Bray–Curtis distance among seasonal groups.

Groups	R^2^	*p*-Value	Significance
Spr vs. Sum	0.096274	0.069	
Spr vs. Fal	0.123058	0.004	**
Spr vs. Win	0.099051	0.023	*
Sum vs. Fal	0.098949	0.04	*
Sum vs. Win	0.092153	0.248	
Fal vs. Win	0.105864	0.005	**
Spr vs. Sum vs. Fal vs. Win	0.146528	0.001	***

R^2^ indicates the proportion of variance explained by group differences. *p*-values show the significance of these differences, assessed by permutation tests. Significance levels: * *p* < 0.05; ** *p* < 0.01; *** *p* < 0.001.

## Data Availability

The data in this study have been deposited in the NCBI SAR under accession number PRJNA1335193.

## Supplementary Materials

The following supporting information can be downloaded at: https://www.mdpi.com/article/10.3390/pathogens14121237/s1, Table S1: Summary of metagenomic sequencing data for the 24 François’ langur fecal samples.
