# Supplementary material for: Seasonal Metagenomic Survey of Pathogenic Microorganisms in Non-Human Primates in Mayanghe National Nature Reserve, China
Source: Pathogens. 2025 Dec 4;14(12):1237. doi: 10.3390/pathogens14121237 (PMC12735446; doi:10.3390/pathogens14121237)
Supplement: Supplementary file 1 [file pathogens-14-01237-s001.zip › pathogens-3975553-supplementary.pdf]

Table S1. Summary of metagenomic sequencing data for the 24 François' langur fecal samples.

Sample_Lib	Raw_reads	Clean_reads	Q20	Q30	Valid_rate
Sum_4_2	32789649 (32.79 M)	32684150 (32.68 M)	98.00%	95.00%	99.68
Sum_4_1	32789649 (32.79 M)	32684150 (32.68 M)	99.00%	97.00%	99.68
Win_5_2	35103186 (35.10 M)	35007954 (35.01 M)	99.00%	96.00%	99.73
Win_5_1	35103186 (35.10 M)	35007954 (35.01 M)	99.00%	98.00%	99.73
Fal_4_2	36630838 (36.63 M)	36534127 (36.53 M)	99.00%	96.00%	99.74
Fal_4_1	36630838 (36.63 M)	36534127 (36.53 M)	99.00%	97.00%	99.74
Spr_6_2	36720150 (36.72 M)	36609244 (36.61 M)	98.00%	95.00%	99.7
Spr_6_1	36720150 (36.72 M)	36609244 (36.61 M)	99.00%	97.00%	99.7
Fal_3_2	37221083 (37.22 M)	37123797 (37.12 M)	98.00%	96.00%	99.74
Fal_3_1	37221083 (37.22 M)	37123797 (37.12 M)	99.00%	98.00%	99.74
Win_2_2	37238919 (37.24 M)	37127869 (37.13 M)	98.00%	95.00%	99.7
Win_2_1	37238919 (37.24 M)	37127869 (37.13 M)	99.00%	97.00%	99.7
Win_3_2	37776674 (37.78 M)	37660175 (37.66 M)	98.00%	95.00%	99.69
Win_3_1	37776674 (37.78 M)	37660175 (37.66 M)	99.00%	98.00%	99.69
Win_4_2	37801932 (37.80 M)	37689916 (37.69 M)	98.00%	95.00%	99.7
Win_4_1	37801932 (37.80 M)	37689916 (37.69 M)	99.00%	98.00%	99.7
Sum_3_2	37802835 (37.80 M)	37717229 (37.72 M)	99.00%	97.00%	99.77
Sum_3_1	37802835 (37.80 M)	37717229 (37.72 M)	99.00%	97.00%	99.77
Win_6_2	38405120 (38.41 M)	38265018 (38.27 M)	98.00%	95.00%	99.64
Win_6_1	38405120 (38.41 M)	38265018 (38.27 M)	99.00%	97.00%	99.64
Sum_6_2	38633062 (38.63 M)	38511088 (38.51 M)	98.00%	95.00%	99.68
Sum_6_1	38633062 (38.63 M)	38511088 (38.51 M)	99.00%	97.00%	99.68
Fal_6_2	40228724 (40.23 M)	40130011 (40.13 M)	99.00%	96.00%	99.75
Fal_6_1	40228724 (40.23 M)	40130011 (40.13 M)	99.00%	97.00%	99.75
Spr_4_2	40405369 (40.41 M)	40285773 (40.29 M)	98.00%	96.00%	99.7
Spr_4_1	40405369 (40.41 M)	40285773 (40.29 M)	99.00%	97.00%	99.7
Spr_5_2	40808900 (40.81 M)	40687680 (40.69 M)	99.00%	96.00%	99.7
Spr_5_1	40808900 (40.81 M)	40687680 (40.69 M)	99.00%	97.00%	99.7
Fal_5_2	41884807 (41.88 M)	41793552 (41.79 M)	99.00%	96.00%	99.78
Fal_5_1	41884807 (41.88 M)	41793552 (41.79 M)	99.00%	98.00%	99.78
Fal_1_2	42916915 (42.92 M)	42813455 (42.81 M)	99.00%	98.00%	99.76
Fal_1_1	42916915 (42.92 M)	42813455 (42.81 M)	99.00%	98.00%	99.76
Spr_1_2	43435795 (43.44 M)	43304456 (43.30 M)	99.00%	96.00%	99.7
Spr_1_1	43435795 (43.44 M)	43304456 (43.30 M)	99.00%	97.00%	99.7
Sum_5_2	44418437 (44.42 M)	44298382 (44.30 M)	98.00%	96.00%	99.73
Sum_5_1	44418437 (44.42 M)	44298382 (44.30 M)	99.00%	97.00%	99.73
Spr_3_2	45531114 (45.53 M)	45419116 (45.42 M)	99.00%	97.00%	99.75
Spr_3_1	45531114 (45.53 M)	45419116 (45.42 M)	99.00%	98.00%	99.75
Sum_1_2	47208445 (47.21 M)	47106138 (47.11 M)	99.00%	97.00%	99.78
Sum_1_1	47208445 (47.21 M)	47106138 (47.11 M)	99.00%	97.00%	99.78
Win_1_2	47610129 (47.61 M)	47498984 (47.50 M)	99.00%	97.00%	99.77
Win_1_1	47610129 (47.61 M)	47498984 (47.50 M)	99.00%	98.00%	99.77

Spr_2_2	47759089 (47.76 M)	47640533 (47.64 M)	99.00%	97.00%	99.75
Spr_2_1	47759089 (47.76 M)	47640533 (47.64 M)	99.00%	98.00%	99.75
Fal_2_2	51674242 (51.67 M)	51557559 (51.56 M)	99.00%	97.00%	99.77
Fal_2_1	51674242 (51.67 M)	51557559 (51.56 M)	99.00%	98.00%	99.77
Sum_2_2	54205296 (54.21 M)	54084837 (54.08 M)	99.00%	97.00%	99.78
Sum_2_1	54205296 (54.21 M)	54084837 (54.08 M)	99.00%	98.00%	99.78
